# Deep convolutional neural networks for automated scoring of pentagon copying test results

**DOI:** 10.1038/s41598-022-13984-7

**Published:** 2022-06-14

**Authors:** Jumpei Maruta, Kentaro Uchida, Hideo Kurozumi, Satoshi Nogi, Satoshi Akada, Aki Nakanishi, Miki Shinoda, Masatsugu Shiba, Koki Inoue

**Affiliations:** 1 Medical Center for Dementia, Osaka City Kosaiin Hospital, 6-2-1, Furuedai, Suita-shi, Osaka Prefecture 565-0874 Japan; 2 Department of Neuropsychiatry, Osaka Metropolitan University Graduate School of Medicine, Osaka, Japan; 3 Osaka Metropolitan University Graduate School of Human Life and Ecology, Osaka, Japan; 4 Center for Brain Science, Osaka Metropolitan University Graduate School of Medicine and Faculty of Medicine, Osaka, Japan

**Keywords:** Psychology, Neurological manifestations, Information technology, Neurological disorders

## Abstract

This study aims to investigate the accuracy of a fine-tuned deep convolutional neural network (CNN) for evaluating responses to the pentagon copying test (PCT). To develop a CNN that could classify PCT images, we fine-tuned and compared the pre-trained CNNs (GoogLeNet, VGG-16, ResNet-50, Inception-v3). To collate our training dataset, we collected 1006 correct PCT images and 758 incorrect PCT images drawn on a test sheet by dementia suspected patients at the Osaka City Kosaiin Hospital between April 2009 and December 2012. For a validation dataset, we collected PCT images from consecutive patients treated at the facility in April 2020. We examined the ability of the CNN to detect correct PCT images using a validation dataset. For a validation dataset, we collected PCT images (correct, 41; incorrect, 16) from 57 patients. In the validation testing for an ability to detect correct PCT images, the fine-tuned GoogLeNet CNN achieved an area under the receiver operating characteristic curve of 0.931 (95% confidence interval 0.853–1.000). These findings indicate that our fine-tuned CNN is a useful method for automatically evaluating PCT images. The use of CNN-based automatic scoring of PCT can potentially reduce the burden on assessors in screening for dementia.

## Introduction

In clinical psychology, tests in which patients copy geometric or representational figures are widely used for detecting and evaluating constructional apraxia. The Mini-Mental State Examination (MMSE), a dementia screening tool, widely used in Japan, includes the Pentagon Copying Test (PCT) as an assessment of constructional apraxia^1^. In the context of the MMSE, the PCT images can dichotomously be assessed as correct or incorrect.

Figure copying tests can lead to biased scoring by different raters; there are attempts to standardize scoring methods in various ways^2–4^. There is also the problem of the human cost of evaluation. Computerized scoring of figure copying tests can be considered reliable because the rater experience does not affect the scoring. A recent study reported the robustness of automated quantitative scoring of PCT has been based on information, such as the number or coordinates of pentagons, obtained from object (or feature) detection^5,6^. However, since patients with dementia often redraw figures many times, or sometimes copy in close proximity to a model figure^7^, there is a possibility that detection may not be successful due to many artifacts. Although Folstein's criterion seems clear at first glance, it may not be sufficient in some cases. Examples of difficult judgement include: (1) the extent to which a slightly rounded corner of a pentagon is acceptable as a corner, and (2) the extent to which a distorted edge of a pentagon is acceptable as a line segment. The PCT images made by patients with suspected dementia vary significantly, and these problems are often experienced in the scoring process. Therefore, it is necessary to create an automatic scoring artificial intelligence (AI) system that has learned the results of scoring made by clinical psychologists as training data.

Recent advances in AI technology may facilitate the automatic scoring of figure copying tests. In recent years, it has been shown that vision task results can readily be assessed with deep learning technologies^8,9^, especially those involving convolutional neural networks (CNN)^10^. A noteworthy advantage of CNNs is that they can be generalized to recognize tasks other than the one for which they were originally designed^11,12^. However, CNNs also have some serious disadvantages. For example, a CNN must be trained with a largely labeled image dataset to avoid overfitting; further, training a CNN from scratch requires a considerable amount of time and computational power. One way to overcome these challenges in creating a CNN is to use fine-tuning to create one to classify specific objects or figures based on a CNN trained to classify natural images^13^.

However, Li et al. reported that they could not achieve sufficient accuracy in PCT correctness using fine-tuned Inception-v3 CNN^6^. They used 658 PCT images (correct 327, incorrect 331) as their training set. The inclusion of the larger number of PCT images from patients suspected with dementia may further improve the accuracy of CNN-based PCT decisions. In this study, we used fine-tuning strategy to create a CNN for automatically evaluating PCT images with the larger number of the training data from patients suspected with dementia and then investigated the accuracy of our CNN.

## Materials and methods

### Ethics statement

The study protocol was approved by the ethics committees of the Osaka City Kosaiin Hospital and the Osaka City University Graduate School of Medicine in accordance with the Declaration of Helsinki (2013) and the Ethical Guidelines for Medical and Biological Research Involving Human Subjects in Japan. Since this study was an observational study using information obtained in routine medical care; no additional tests or questionnaires were conducted for this study, informed consent was waived by the ethics committees of the Osaka City Kosaiin Hospital and the Osaka City University Graduate School of Medicine. The patients whose PCT images were used were given the opportunity to opt out of the study through an online or offline application. Failure to opt out was regarded as giving consent for the use of their PCT images, demographic data, and psychological test data.

### Datasets

For a training dataset, we retrospectively collected 1006 correct PCT images and 758 incorrect PCT images from dementia suspected patients who underwent treatment at Osaka City Kosaiin Hospital (Osaka, Japan) between April 2009 and December 2012.

Patients who visited Osaka City Kosaiin Hospital in April 2020 and underwent PCT as routine medical care of their regular medical care were included as validation participants. The validation dataset comprised PCT images created by the participants.

The invitation to participate in the study was posted on the hospital bulletin board and the website of the Osaka City University Graduate School of Medicine, our collaborating institution. None of the participants wished to opt out.

### PCT procedures

The PCT was administered to the patients by clinical psychologists during routine care. In the procedure, the patients were asked to copy an image of two intersecting pentagons with pencils on blank sheets of paper. The drawings were then scanned with a SCANSNAP iX500 scanner (Fujitsu, Tokyo, Japan), and the scanned images were cropped to focus on the drawings of the pentagons. If nothing was drawn, then a blank area was cropped. Psychologists classified each PCT drawing as correct or incorrect based on Folstein’s MMSE criteria, which defines a correct PCT drawing as being “composed of two overlapping pentagons, with the overlapping shape being a rhombus”^1,14^. If there is any doubt about the scoring, multiple psychologists consult with each other to standardize the scoring criteria.

### Fine-tuning

To create a CNN capable of classifying PCT images, we fine-tuned the CNNs (GoogLeNet, VGG-16, ResNet-50, Inception-v3) based on the training dataset PCT images. We used the Deep Learning Toolbox in MATLAB 2021b (MathWorks, Natick, MA, USA) for all data augmentation and fine-tuning procedures.

In each CNN, the last fully connected layer was replaced with a new fully connected layer with two classes (correct, incorrect). For initial data augmentation, the training dataset PCT images were randomly shifted (− 10 to 10 pixels), resized (0.7 to 1.0), and rotated (− 90° to 90°). An optimization algorithm called stochastic gradient descent with momentum was used as a solver for training the CNNs. The solver parameters were as follows: mini-batch size, 32; maximum epochs, 200; and initial learning rate, 0.0003. For each PCT image, the last layer was arranged to output a value for a variable called “probability of PCT correct,” hereafter abbreviated as “P(PCTcorrect).” The P(PCTcorrect) expressed the CNN’s estimate for the probability that a given PCT image had been categorized as correct. The values of the P(PCTcorrect) variable ranged from 0 to 1, with higher values indicating greater CNN-calculated probabilities that a given PCT image had been categorized as correct.

### Validation testing

The fine-tuned CNNs were used to calculate a P(PCTcorrect) value for each validation dataset PCT image. The performance of P(PCTcorrect) value for each CNN was evaluated in terms of the following performance metrics: (1) accuracy, (2) precision, (3) recall (sensitivity), (4) specificity, (5) area under the receiver operating characteristic curve (AUROC). The AUROC was used as an indicator for comparison between the fine-tuned CNNs. Here, true positive (TP) denotes correctly copied PCT images classified as correctly copied ones when P(PCTcorrect) was above the optimal threshold. True negative (TN) denotes incorrectly copied PCT images classified as incorrectly copied ones when P(PCTcorrect) was not above the optimal threshold. False positive (FP) denotes incorrectly copied PCT images classified as correctly copied ones. False negative (FN) denotes correctly copied PCT images classified as incorrectly copied ones.

1 $$accuracy = \frac{TP + TN}{{TP + FP + TN + FN}}$$

2 $$precision = \frac{TP}{{TP + FP}}$$

3 $$recall = \frac{TP}{{TP + FN}}$$

4 $$specificity = \frac{TN}{{TP + FP + TN + FPN}}$$

### Statistical analysis

All statistical analyses were performed with Easy R (Saitama Medical Center, Jichi Medical University, Saitama, Japan)^15^, which is a graphical user interface implemented in R version 2.13.0 (R Foundation for Statistical Computing, Vienna, Austria). We set our statistical significance threshold at *p* < 0.05. The confidence interval for each area-under-the-curve (AUC) value was calculated with the DeLong test. Optimal cutoff threshold was determined at the closest point to the upper left corner.

## Results

The PCT images drawn by 57 patients were collected as a validation data set. The patients’ backgrounds were as follows: 37 females, 20 males, mean age 78.16 ± 10.76 years old, 39 patients with Alzheimer’s disease, 4 patients with psychiatric disorders, 4 with frontotemporal dementia, 3 patients with cerebrovascular dementia, and 7 with other diseases. Of the 57 PCT images for validation, 41 were correctly copied by Folstein’s MMSE criteria.

Table 1 shows the performance metrics of the fine-tuned CNNs in validation testing. The finetuned GoogLeNet CNN achieved the highest AUROC.

**Table 1:** The performance metrics of CNN models for the validation dataset images.

CNN model	Accuracy	Precision	Recall (sensitivity)	Specificity	AUROC
GoogLeNet	0.877	0.947	0.878	0.875	0.931
VGG-16	0.930	0.951	0.951	0.875	0.922
ResNet-50	0.789	0.872	0.829	0.688	0.784
Inception-v3	0.789	0.939	0.756	0.875	0.864

AUROC, area under the receiver operating characteristic curve; CNN, convolutional neural network.

**Figure 1:**
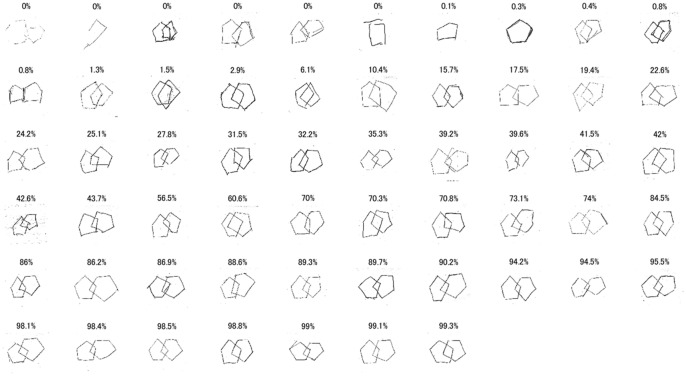
The validation dataset images and the P(PCTcorrect) values calculated by the fine-tuned GoogLeNet CNN. CNN, convolutional neural network; PCT, pentagon copying test; P(PCTcorrect), CNN-calculated probability of the PCT image being categorized as correct.

Figure 1 shows the PCT images in validation dataset and the P(PCTcorrect) values calculated by the fine-tuned GoogLeNet CNN. The lower the value of P(PCTcorrect), the more the copy tended to collapse. A fine-tuned GoogLeNet CNN embedded in an iPhone application is available for download (Available on the Testflight: https://testflight.apple.com/join/xXmo7rRi).

**Figure 2:**
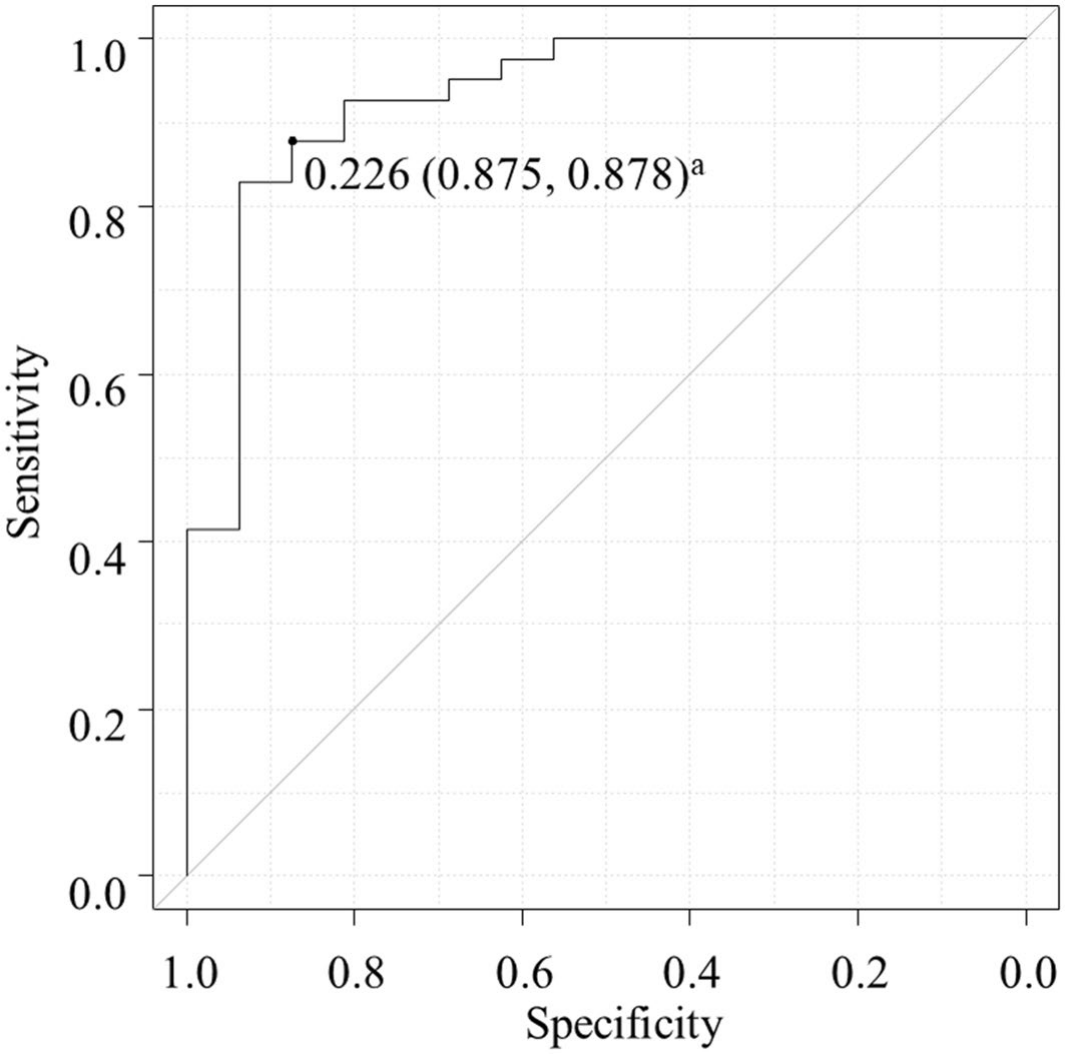
ROC curve in the validation dataset for prediction of the PCT images being categorized as correct based on P(PCTcorrect) values calculated by the fine-tuned GoogLeNet CNN. ^a^The cut-off probability (specificity, sensitivity) is shown at the point closest to the top left-hand corner. CNN, convolutional neural network; PCT, pentagon copying test; P(PCTcorrect), CNN-calculated probability of the PCT image being categorized as correct; ROC, receiver operating characteristic.

Figure 2 shows the ROC curve in the validation dataset for prediction of the PCT images being categorized as correct based on P(PCTcorrect) values calculated by the fine-tuned GoogLeNet CNN. The area under the receiver operating characteristic curve (AUROC) was 0.931 (95% confidence interval 0.853–1.000).

## Discussion

In this study, we fine-tuned pre-trained CNNs using the larger number of patient's PCT images than the previous study and the clinical psychologist's scoring results as training data. Furthermore, we also collected time-separated validation data to evaluate the accuracy of the CNNs. The P(PCTcorrect) value calculated by the fine-tuned GoogLeNet CNN was in strong agreement with the scoring results by clinical psychologists. Although the tested data sets are different and cannot be simply compared, the AUROC of the P(PCTcorrect) of the fine-tuned GoogLeNet CNN, 0.931, had outperformed the AUROC of the CNN using supervised transfer learning reported by Li et al.^6^, 0.72, and was the highest among the automatic scoring of PCTs using fine-tuning strategy.

Our findings indicate that our fine-tuned GoogLeNet CNN may be useful for automatically evaluating PCT images. The P(PCTcorrect) value agrees with PCT correct with high accuracy, which may be useful for scoring PCT images. The P(PCTcorrect) is useful as a reference to evaluate the constructional apraxia. The results of this study suggest using AI to assess constructional apraxia.

Fine-tuned CNN may also be useful in the assessment of figure copying tests other than the PCT. In this study, we were able to fine-tune a pre-trained CNN for PCT scoring without any special adjustments just by preparing the teacher data. Contrarily, in the study that evaluated PCTs using object or feature detection, it was necessary to set up a system to detect the features of PCTs that would be scored as correct answers^5,6^. The usefulness of fine-tuned CNN to evaluate the clock drawing test and the Rey-Osterrieth complex figure copying test has also been reported^16,17^.

Of the multiple CNNs compared, the fine-tuned GoogLeNet CNN achieved the highest AUROC. The GoogLeNet CNN had relatively low ImageNet validation accuracy among the CNNs compared^18^. This may be due to the fact that PCT images are very different from the natural images (dogs, boats, etc.) targeted by ImageNet.

Further, our CNN resulted in incorrect scoring for some images. The cause of the mistakes was unknown because the features captured by CNN to make its decisions were unknown. This is a common problem in AI implemented by deep learning^19^.

This study has several limitations. First, the validation participants did not include any patients with finger tremors. As per Folstein’s guidelines for the MMSE, tremors should be ignored when scoring various test results, but it is often difficult to evaluate PCT images for patients with finger tremors. Building on the findings of this study, future studies should examine the robustness of our CNN when evaluating PCT images from patients with finger tremors.

This study also has certain strengths. In the present study, a fine-tuned CNN based on pre-trained GoogLeNet CNN automatically scored the PCT images; the results were in high agreement with the results obtained by clinical psychologists using Folstein’s MMSE criteria (AUROC, 0.931). The automatic scoring of PCTs using the CNN presented here does not require any input using mobile devices and removing artifacts. Therefore, there are fewer restrictions for conducting the test. An automatic PCT scoring using CNNs may reduce the burden and assessment bias of raters in dementia screening.

## Data Availability

The datasets generated during and/or analyzed during the current study are available from the corresponding author on reasonable request.
